# Response of mid-lactation primiparous Holstein cows to the supplementation of rumen-protected methionine during the summer

**DOI:** 10.1371/journal.pone.0343747

**Published:** 2026-02-26

**Authors:** Caio R. Monteiro, Victor Augusto de Oliveira, Rabeche Schmith, João Pedro A. Rezende, Tales L. Resende, João A. Negrão, Marina A. C. Danés

**Affiliations:** 1 Department of Animal Science, Federal University of Lavras, Lavras, Minas Gerais, Brazil; 2 Evonik Brasil Ltda., São Paulo, São Paulo, Brazil; 3 Department of Basic Sciences, Faculty of Animal Science and Food Engineering (FZEA), University of São Paulo (USP), Pirassununga, São Paulo, Brazil; Universitas Sebelas Maret, INDONESIA

## Abstract

This study aimed to evaluate the effects of rumen-protected methionine (RPM) supplementation on productive and physiological responses of primiparous Holstein cows during summer. We hypothesized that RPM supplementation would maintain or improve milk yield and composition due to beneficial physiological, redox, and inflammatory responses in cows exposed to summer heat. The trial was conducted in a randomized block design during nine weeks in Brazil using 80 primiparous cows (182 ± 64 DIM; 42.9 ± 4.7 kg/d milk). Cows were blocked by milk yield and DIM and assigned to a control diet (CON; no added RPM) or the same diet supplemented with RPM (Mepron®, Evonik) at 0.75 g/kg diet dry matter, targeting 20 g/cow/day (product contains 62% metabolizable methionine) to the average cow. Milk yield and composition, vaginal temperature, respiratory rate, and plasma samples were collected in weeks 3, 6, and 9. Data were analyzed using mixed models including treatment, week, and their interaction as fixed effects, and block and cow as random effects. Cows were maintained under naturally occurring summer conditions. Environmental monitoring during weeks 3, 6, and 9 indicated elevated temperature–humidity index (THI) values, with values remaining above the heat-stress threshold (THI > 68) for 68.3% of the monitored hours (mean THI = 70.6; range 61.0–84.4). Overall (least squares mean across weeks 3, 6, and 9), RPM increased milk yield by 2.0 kg/d (44.9 vs. 42.9 kg/d), protein yield by 50 g/d (1,464 vs. 1,414 g/d), lactose yield by 108 g/d (2,109 vs. 2,001 g/d), and total solids yield by 176 g/d (5,331 vs. 5,155 g/d). Lactose concentration was lower in RPM (4.71 vs. 4.76%). Fat yield was unaffected, but a treatment × week interaction was observed for fat content. Milk fatty acid (FA) profile was unchanged, although treatment × week interactions were observed for individual fatty acids (C16:0, C18:0, C18:1, and preformed FA). Plasma glucose was lower, and insulin was higher in RPM than in CON cows (39.3 vs. 43.2 mg/dL and 0.52 vs. 0.35 ng/mL, respectively). Antioxidant capacity improved, with RPM cows having greater ferric reducing antioxidant power (32.9 vs. 28.5 µM) and lower malondialdehyde (2.48 vs. 2.78 nmol/mL). Other biochemical, inflammatory, and immune markers were unaffected. Respiratory rate was slightly higher in RPM than in CON cows (55 vs. 50 breaths/min). Mean vaginal temperature did not differ between treatments; however, a treatment × time × hour interaction was observed. Supplementation with RPM improved milk and solids yield, and enhanced antioxidant capacity and insulin levels, supporting its use to improve metabolic resilience under warm conditions.

## Introduction

Heat stress (HS) is a well-recognized challenge that negatively affects the performance, health, and reproduction of dairy cows. Elevated temperatures are also associated with higher incidence of metabolic and infectious diseases and with impaired fetal development, potentially affecting future productive performance [1,2]. The ongoing global warming trend has intensified this issue, with climate models projecting a rise of approximately 0.2°C per decade in global mean temperature [3].

Although reduced dry matter intake (DMI) is considered the primary factor contributing to production losses during HS, it accounts for 35–50% of the observed decline in milk yield [4]. Additional physiological mechanisms, such as systemic inflammation, immune activation, and changes in the antioxidant system, contribute to the disruption of metabolic homeostasis [5,6]. These mechanisms alter nutrient partitioning, reducing the availability of key substrates for productive functions and increasing the diversion of amino acids (AA) toward nonproductive uses in the liver and gastrointestinal tract [7,8].

Physical microclimate modifications (e.g., shade, increased air movement using fans, and evaporative cooling or cow-wetting strategies such as sprinklers/soakers or spray/misting) are widely adopted to enhance heat dissipation and mitigate heat stress in dairy cows [9–11]. In parallel, nutritional strategies aimed at ameliorating the negative effects of heat stress have been investigated, but they are still less extensively characterized than mechanical cooling methods. These approaches include fat supplementation [12,13], vitamin and mineral supplementation [14,15], plant polyphenol extracts [16], antioxidant blends [17], and amino acid supplementation [18–20].

Among these, amino acids (AA) supplementation has emerged as a promising approach to support antioxidant capacity, modulate immune function, and maintain lactational performance. Methionine (Met) is frequently the most deficient AA in dairy cow diets and plays a central role not only in milk protein synthesis but also in one-carbon metabolism [21]. Under stress conditions, methionine is particularly important due to its involvement in methylation reactions, glutathione synthesis, and immune modulation [22].

Supplementation with rumen-protected methionine (RPM) during the transition period (from 3 wk prepartum to 3 wk postpartum) has been associated with improved lactational performance in multiparous Holstein cows. Feeding rumen-protected Met from −21 d to 30 DIM at 0.07–0.19% of diet DM increased milk yield by 3.4 kg/d and milk protein concentration by 0.18 percentage units [23], whereas ethyl-cellulose RPM fed from −28–60 d relative to parturition at 0.09–0.10% of diet DM increased milk yield by 4.1 kg/d and milk protein concentration by 0.16 percentage units during 1–30 DIM [24].

Continuous exposure to heat stress (HS) in dairy cows for one [25] to four [26] weeks can have cumulative negative impacts on production. In contrast, most studies evaluating rumen-protected methionine (RPM) under HS have relied on short-term challenge models lasting only a few days (e.g., a 9-d HS challenge), and therefore evidence on RPM supplementation during naturally occurring summer HS lasting several weeks, particularly in primiparous cows, remains limited. This knowledge gap is relevant because primiparous cows may be moresusceptible under HS due to the simultaneous demands of growth, lactation, and metabolic adaptation [27,28].

Therefore, the objective of this study was to evaluate the effects of RPM supplementation on productive and physiological responses of primiparous Holstein cows during the summer. We hypothesized that, despite both groups being managed under the same mechanical/evaporative cooling system, RPM supplementation would maintain or improve milk yield and composition, as well as support beneficial physiological, redox, and inflammatory responses in lactating cows exposed to naturally occurring summer conditions in Brazil.

## Materials and methods

### Ethics statement

All procedures were approved by the Ethics Committee on the Use of Animals (CEUA) of the Federal University of Lavras under protocol number 056/22.

### Location, animals, management and facilities

The study was conducted on a commercial farm located in Três Corações, Minas Gerais, in the Southeast region of Brazil, from December 2022 to February 2023. The farm is situated at 910 m above sea level, at latitude 21°44’59.22“S and longitude 45°11’25.64”W.

A total of 80 primiparous Holstein cows with an average of 182 ± 64 days in milk (DIM), producing 42.9 ± 4.7 kg of milk per day, with 2.93 ± 0.65% milk fat and 3.13 ± 0.27% milk protein (mean ± SD) at the beginning of the experimental period were used. The animals were housed in a compost barn with 120 other non-experimental cows, totaling 200 cows. They were milked three times daily at 04:00, 12:00, and 20:00 in a herringbone parlor with automatic detachers.

The animals were housed in two pens measuring 16 × 50 m (800 m²) each, with 100 animals per pen (40 experimental and 60 non-experimental cows), resulting in a stocking density of 8.0 m² per cow. Each pen was assigned to one dietary treatment and was fed separately under commercial farm conditions; therefore, individual daily dry matter intake was not measured. Bedding consisted of wood shavings, mechanically turned twice daily. Both pens were equipped with four fans per pen (Mamute F200; FrontAgro Ltda., São José do Rio Preto, SP, Brazil) operating continuously and a sprinkler line along the feed bunk running on a 30 s on/4 min off cycle. The cooling system was managed identically in both pens throughout the experimental period.

### Diets and experimental design

The experimental design was a randomized complete block design with two treatments. The 80 experimental cows were organized into 40 blocks (pairs) based on pre-trial milk production and DIM. Within each block, cows were randomly assigned to one of two treatments (one cow per treatment within each block): a control diet (CON) or CON plus the inclusion of RPM (Mepron^®^, Evonik Operations GmbH, Hanau, Germany), encapsulated with ethyl cellulose. At enrollment, cows were allocated to their respective pens and remained in the same pen throughout the experimental period.

A total of 40 cows per treatment were used, except for vaginal temperature, which was measured in a subsample of 15 cows per treatment. Sample size was determined using milk protein yield as the primary response variable, following Kononoff and Hanford [29] and adapted to the present design. Calculations were performed using a two-sided α = 0.05 with a target power of 0.80. Based on farm records collected before the start of the experiment, the standard deviation of milk protein yield was 94 g/d, and a 40 g/d difference was considered the minimum relevant effect to detect.

Experimental diets (Table 1) were formulated to meet or exceed requirements for metabolizable energy, metabolizable protein, vitamins, and macrominerals, as recommended by NASEM [30], to support milk production. The RPM dose was 0.75 g/kg of dietary dry matter (DM) in the total mixed ration (TMR; 0.075% of DM), corresponding to approximately 0.47 g of metabolizable methionine per kg of diet DM (≈0.047% of DM), based on 62% metabolizable methionine in the product, and to approximately 13 g of metabolizable methionine per cow per day. This dose was selected to increase the estimated metabolizable methionine supply and resulted in a methionine utilization efficiency below the target, indicating a nonlimiting (adequate-to-excess) supply of metabolizable methionine according to the NASEM ration evaluation [30]. The RPM product was pre-mixed into the mineral and vitamin supplement included in the TMR. The control diet contained no added rumen-protected methionine (0 g/kg DM of RPM). Baseline methionine supply was provided by the basal ingredients in both diets. Estimated metabolizable methionine supply and the Lys:Met ratio are shown in Table 1.

**Table 1:** Ingredient and nutrient composition of control (CON) and rumen-protected methionine (RPM) supplemented diets¹.

	CON	RPM
*Ingredient, % of DM*		
Corn silage, main crop	41.0	41.0
Corn silage, second crop	11.1	11.1
Soybean meal	16.9	16.9
Citrus pulp	8.9	8.9
Corn, high-moisture silage	7.6	7.6
Distillers’ dried grains	4.4	4.4
Pelletized soybean hulls	2.3	2.3
Cottonseed	4.2	4.2
Palm fat^2^	1.0	1.0
PREMIX^3^	2.5	2.5
Rumen-protected methionine^4^	---	0.075
*Chemical composition, % of DM*		
Crude protein	17.4	17.5
Metabolizable protein	10.0	10.1
Rumen-degradable protein	11.5	11.5
Rumen-undegradable protein	5.9	6.0
Starch	24.7	24.7
Neutral detergent fiber	29.3	29.3
Acid detergent fiber	17.7	17.7
Fatty acids	2.8	2.8
Ash	4.4	4.4
Metabolizable methionine, g/day^5^	55.0	68.0
Diet Lys:Met ratio^5^	3.2	2.5

^1^Diets were formulated to be isoenergetic and isonitrogenous.

^2^Palm fat: GoldenFat® (CSB Indústria e Comércio Ltda., Bragança Paulista, São Paulo, Brazil), composed of calcium salts of palm fatty acids. Guaranteed analysis: ether extract, minimum 700 g/kg; ash, maximum 190 g/kg; calcium, 100–110 g/kg.

^3^PREMIX: PEC-PRO Lactation Top 700 ORG (PEC-MIX Suplementos Minerais Ltda., Alfenas, Minas Gerais, Brazil). Guaranteed analysis: 9–14% Ca; 2.4% P; 11.4% Na; 3.1% Mg; 0.7% S; 1.7% K; 0.3% Zn; 0.2% Mn; 503 mg/kg Cu; 37 mg/kg I; 31.1 mg/kg Co; 17.8 mg/kg Se; 9.8 mg/kg Cr. Vitamins: A (220,657 IU/kg), D₃ (70,560 IU/kg), E (1,844 IU/kg). Additives: Monensin (475 mg/kg), Virginiamycin (490 mg/kg), Biotin (49 mg/kg), Bacillus subtilis (30 × 10¹¹ CFU/kg), β-glucans (3,160 mg/kg), glucomannans (5,080 mg/kg), mannan-oligosaccharides (1,920 mg/kg), and calcium/sodium aluminosilicate (7,920 mg/kg).

^4^Rumen-protected methionine: Mepron® (Evonik Operations GmbH, Hanau, Germany), a rumen-protected DL-methionine product containing 85% DL-methionine.

^5^Estimated using NASEM (2021).

The experimental diets were provided as a TMR once daily at 08:00. Ingredients were weighed and mixed in a mixer wagon with an integrated scale (Siloking DUO 14-T®, Siloking do Brasil Ltda., São José do Rio Preto, São Paulo, Brazil). Refusals were weighed daily at the pen level, and the TMR amount was adjusted based on the average pen refusals from the previous two days, targeting 5% refusals. Animals had *ad libitum* access to TMR and fresh water. The TMR was pushed up with a motorized blade at least 10 times per day. Refusals were used only to manage feed delivery and were not used to estimate individual dry matter intake.

Due to the nature of the experimental design, blinding was not possible. The research team was aware of group allocation during animal assignment and the conduct of the experiment. Farm staff and researchers responsible for feeding and sampling were also aware of treatments. For standardization, the 120 non-experimental cows were also paired by parity, milk production, and DIM and randomly assigned to one of the two pens. Thus, each pen contained 100 cows (40 experimental/sampled and 60 non-experimental/non-sampled) and was randomly assigned to one of the two treatments (CON or RPM).

### Data and sample collection and analysis

Before the start of the experiment and during experimental weeks 3, 6, and 9, TMR ingredients were sampled separately once daily. A weekly composite sample of each ingredient was stored frozen until the end of the trial. After completion of the experiment, composite samples were dried in a forced-air oven at 55°C for 72 h and ground to 1 mm in a Willey-type mill for analysis of dry matter [31], organic matter [31], ash [32], crude protein [32], ether extract [31], neutral detergent fiber (NDF) with amylase and sodium sulfite [33], acid detergent fiber [34], and starch [35]. Analyses were conducted at UFLA’s Animal Production Laboratory, in Lavras, Minas Gerais, Brazil. The mean nutritional composition of the diet is presented in Table 1.

The trial lasted 9 weeks (63 days). The sampling weeks were defined as days 14–21 (week 3), 35–42 (week 6), and 56–63 (week 9) relative to the start of the experimental diets. During each sampling week, milk yield was recorded daily. Individual milk samples were collected on two consecutive days (days 19–20, 40–41, and 61–62, corresponding to the third-to-last and second-to-last day of each sampling week), and daily composite samples were prepared proportionally to the yield of each milking. Blood samples were collected on the last day of each sampling week (days 21, 42, and 63). Respiratory rate was recorded on two consecutive days during each sampling week (days 19–20, 40–41, and 61–62). Vaginal temperature was recorded continuously over four consecutive days corresponding to the last four days of each sampling week (days 18–21, 39–42, and 60–63). Mean (± SD) DIM on the last day of each sampling week (days 21, 42, and 63) was 203.6 ± 68.0, 224.6 ± 68.0, and 245.6 ± 68.0 for CON cows, and 201.6 ± 59.5, 222.6 ± 59.5, and 243.6 ± 59.5 for RPM cows, respectively.

### Environmental conditions

Air temperature (°C) and relative humidity (%) were recorded automatically at 10-minute intervals inside the compost-barn by four automatic loggers (IP-747RH, Impac Comercial e Tecnologia Ltda., Vargem Grande Paulista, São Paulo, Brazil) positioned throughout the barn (two in each pen), 2 m above the floor. Temperature-humidity index (THI) was calculated at 1-hour intervals according to Mader et al. [36], using the mean ambient temperature and relative humidity recorded during each hour, as follows: THI=(0.8 x T)+[RH x (T−14.4)]+46.4, where T is the ambient temperature (°C), and RH is the relative humidity expressed as a decimal fraction. The percentage of hours per day when THI exceeded 68 and 72 was then calculated. Across the entire experimental period, ambient temperature ranged from 16.1 to 33.0°C and THI ranged from 61.0 to 84.4. Environmental data are summarized in the Results section.

### Vaginal temperature, and respiratory rate

Vaginal temperature (VT; °C) was measured in a subsample of 30 cows (15 per treatment) every five minutes over four consecutive days during weeks 3, 6, and 9 using an automatic temperature recorder (Thermochron iButton DS1922T-F5#, Analog Devices, Inc., Wilmington, MA, USA) with an accuracy of ±0.5°C and a measurement range of −10°C to +65°C. Temperature recorders were attached to an intravaginal device without progesterone (CIDR®; Zoetis, São Paulo, Brazil). Respiratory rate (RR) was measured by counting flank movements over 30 seconds for all experimental cows (n = 40) on two consecutive days during weeks 3, 6, and 9, starting at 10:00 and 17:00.

### Milk collection and analysis of components

Milk production was measured on two consecutive days before the start of the experiment (baseline; days −2 and −1) and was included as a covariate in the statistical analyses of the corresponding outcomes (i.e., baseline milk yield for milk production traits, and baseline milk component values for milk composition traits). Milk production was also recorded daily during sampling weeks 3, 6, and 9 using an automatic meter (ACR Smart MMV, Interplus, Albinea, RE, Italy). Individual milk samples were collected before the start of the experiment (baseline) and during weeks 3, 6, and 9. Sampling was performed on two consecutive days (six milkings) during each sampling week (days 19–20, 40–41, and 61–62 relative to the start of the trial), at each milking (04:00, 12:00, and 20:00). A daily composite sample was prepared proportionally to the yield of each milking for each cow. Milk samples were stored in plastic vials with 2-bromo-2-nitropropane-1,3-diol (bronopol) preservative, homogenized, and kept refrigerated (4°C) until sent for analysis.

Individual daily milk samples were analyzed by mid-infrared Fourier-transform infrared spectroscopy (FTIR; CombiFoss 7, FOSS Analytics, Hillerød, Denmark) to determine fat, protein, lactose, casein, total solids. Milk fatty acids (FA) and FA groups (e.g., SFA, UFA, MUFA, PUFA; and chain-length classes) were predicted from FTIR spectra using the instrument’s FA prediction models. Somatic cell count was determined by flow cytometry (MilkoscanCombiFoss 7, FOSS Analytics, Hillerød, Denmark). Analyses were conducted at the Clínica do Leite Institute Laboratory (Piracicaba, São Paulo, Brazil), without treatment identification. Daily milk solids yield was calculated using the milk yield and composition from the same day of sampling. Energy-corrected milk (ECM) was calculated using the equation: ECM = [0.327 × milk yield (kg)] + [12.95 × fat yield (kg)] + [7.2 × protein yield (kg)], based on the week the milk was sampled [30].

The FTIR-based method quantified specific FA, including C14, C16, C18, C18:1, as well as broader categories such as saturated FA (SFA), unsaturated FA (UFA), monounsaturated FA (MUFA), and polyunsaturated FA (PUFA). Additionally, FA were also grouped according to their origin, comprising short-chain FA (C4 to C14) – de novo; medium chain FA (C15 and C16) – mixed; and long-chain fatty acids (C17 or more) – preformed. Daily yield of each milk fatty acid was calculated by multiplying its concentration by the daily milk yield.

### Blood collection and analysis of metabolites

Blood samples were collected before the start of the experiment as a covariate and once during weeks 3, 6, and 9 by coccygeal vein puncture, always on the last day of the sampling week (days 21, 42, and 63), always after the second milking (starting at 12h15m) and approximately 4 h after feeding (TMR offered once daily at 08:00). Three samples per animal were collected: one for serum collection in a tube with clot activator, and two for plasma collection (one in a tube with sodium heparin anticoagulant and another in a tube with K3 EDTA). Samples were kept on ice and, after collection, centrifuged (3,000 rpm) for 30 minutes at room temperature to obtain serum or plasma and then frozen at −20°C in 2 mL microtubes for later analysis.

Serum samples were analyzed to assess nutritional status and liver health using commercial kits (all from Bioclin-Quibasa, Belo Horizonte, Minas Gerais, Brazil) and an automatic biochemical analyzer (SX-300, Sinnowa Brasil, Ribeirão Preto, São Paulo, Brazil). The analyses included albumin (K040-1), total cholesterol (K083-2), HDL cholesterol (K071-23), glucose (K-082-2,), urea nitrogen (K056-4.1), alanine aminotransferase (ALT; K049-6), and aspartate aminotransferase (AST; K048-6).

Oxidative status was assessed by analyzing plasma concentrations of ferric reducing antioxidant power (FRAP) using a commercial fluorometric kit (MAK509, Sigma-Aldrich, Saint Louis, MO, USA) in samples collected during week 9. Concentrations of malondialdehyde [37], superoxide dismutase [38], and glutathione peroxidase [39] were determined in samples collected during the covariate period and weeks 3, 6, and 9.

Plasma concentrations of non-esterified fatty acids (NEFA) were assessed using an enzyme-linked immunosorbent assay (ELISA; FA115, Randox Laboratories Ltd., Crumlin, County Antrim, UK). Concentrations of haptoglobin and immunoglobulin A (IgA) were determined by polyacrylamide gel electrophoresis [40]. Hormonal response was evaluated using ELISA tests to measure plasma concentrations of insulin (3625-300A, Monobind Inc., Lake Forest, CA, USA) and cortisol (2425-300B, Monobind Inc., Lake Forest, CA, USA). All these analyses were performed using plasma samples collected only during week 9, when environmental temperatures were more challenging. Laboratory analyses performed by external facilities were conducted without treatment identification.

### Statistical analysis

Statistical analyses were conducted using the MIXED procedure in SAS (version 9.4; SAS Institute Inc., Cary, NC, USA). Response variables for blood parameters, milk production, and milk composition were evaluated using a randomized complete block with repeated measures over time, with pre-experiment collection values included as covariates in the model when baseline measurements of the same outcome were available. The model included week of sampling and treatment as fixed effects, and block, experimental unit (cow), and treatment-by-time interaction as random effects, as per the following model:


$$Y_{ijkl}\;=\;\mu \;+\;T_{i}\;+\;COY\;+\;W_{l}\;+\;B_{j}\;+\;V_{k}\left(B\right)\;\;+\;T\;x\;W_{il}\;+\;E_{ijkl}$$

Where: Y ***=*** dependent variable; μ ***=*** overall mean; $T_{i}$ ***=*** fixed effect of treatment (i = CON or RPM); COY ***=*** covariate for Y; $W_{l}\;$ ***=*** fixed effect of week (l = 3, 6 or 9); $B_{j}$ ***=*** random effect of block (j = 1–40); $V_{k}\left(B\right)=$ random effect of the experimental unit nested within block (k = 1–80); $T\;x\;W_{il}$ ***=*** treatment-by-week interaction; and $E_{ijkl}$ ***=*** residual error.

For blood metabolites that were measured only once during the experiment (week 9), such as haptoglobin, immunoglobulin A (IgA), insulin, cortisol, and ferric reducing antioxidant power (FRAP), the analysis was performed without including pre-experiment values as covariates or week as a repeated factor, because baseline values were not available for these analytes.

For respiratory rate evaluation, no covariate was included because pre-experiment (baseline) respiratory rate measurements were not available, and the model had week of sampling, time of day (10:00 and 17:00 h), and treatment as fixed effects, with block, experimental unit (cow), and treatment-by-time interaction as random effects, as per the following model:


$$Y_{ijkl}\;=\;\mu \;+\;T_{i}\;+\;H_{m}\;+\;W_{l}\;+\;B_{j}\;+\;V_{k}\left(B\right)\;\;+\;T\;x\;W_{il}\;+\;T\;x\;H_{im}\;+\;E_{ijklm}$$

Where: Y ***=*** dependent variable; μ ***=*** overall mean; $T_{i}$ ***=*** fixed effect of treatment (i = CON or RPM); $H_{m}$ ***=*** fixed effect time of day (10:00 or 17:00 h); $W_{l}$ ***=*** fixed effect of week (l = 3, 6 or 9); $B_{j}$ ***=*** random effect of block (j = 1–40); $V_{k}\left(B\right)$ ***=*** random effect of the experimental unit nested within block (k = 1–80); $T\;x\;W_{il}$ ***=*** treatment-by-week interaction; $T\;x\;H_{im}$ ***=*** treatment-by-time interaction, and $E_{ijklm}$ ***=*** residual error.

Data normality was assessed using the Shapiro-Wilk test (P < 0.10). When normality assumptions were not met, variables were analyzed using rank transformation (PROC RANK). Rank-transformed variables included milk composition variables expressed as percentages (milk fat, milk protein, lactose, total solids, and casein), somatic cell count, albumin, HDL cholesterol, ALT, AST, ferric reducing antioxidant power (FRAP), non-esterified fatty acids (NEFA), insulin, cortisol, and respiratory rate. P-values were obtained from models fitted on the rank-transformed data, whereas results are presented in the original units for interpretation.

Covariance structures were tested, and the corrected Akaike Information Criterion (AICc) was used to determine the best covariance structure. Studentized residuals were used to detect and remove outliers (≥ 3 or ≤ −3). The Least-squares means (LSMEANS) statement was used for mean comparisons, with Tukey-Kramer adjustment for significant effects. Significance was declared at P ≤ 0.05, and trends were noted at 0.05 < P ≤ 0.10.

All outcomes were recorded at the cow level and analyzed using cow-level repeated-measures models; however, because treatments were applied at the pen level (one pen per treatment), pen-level replication was not available.

## Results

### Environmental conditions

Temperature and relative humidity were recorded inside the compost barn (in-barn microclimate) under the cooling system. During the experimental period, the average ambient temperature was 22.3 ± 1.6 °C, with an average daily maximum of 27.4 ± 2.9 °C and a minimum of 18.8 ± 1.0 °C. Relative humidity averaged 84.7 ± 5.8%, ranging from 41.7 to 100%. The mean temperature-humidity index (THI) was 70.6 ± 2.0, with THI values exceeding 68 and 72 for 68.3% and 32.5% of the total hours, respectively, indicating sustained environmental challenge (Table 2).

**Table 2:** Mean ambient temperature, relative humidity, and temperature-humidity index (THI) during the monitored sampling weeks (weeks 3, 6, and 9) of the 9-week (63-d) trial conducted in lactating primiparous Holstein cows.

Item	Overall (weeks 3, 6, and 9)	Week 3	Week 6	Week 9
THI^1^ > 68, % of time	68.3	56.1	76.5	72.5
THI > 72, % of time	32.5	19.8	37.7	40.1
THI mean	70.6	69.0	71.2	71.6
THI maximum	84.4	84.4	80.1	82.1
THI minimum	61.0	61.0	63.6	64.6
Temperature mean, °C	22.3	21.1	22.7	23.1
Temperature maximum, °C	33.0	31.8	31.8	33.0
Temperature minimum, °C	16.1	16.1	17.6	18.2
Relative humidity mean, %	84.7	88.7	83.3	82.2
Relative humidity maximum, %	100.0	100.0	100.0	100.0
Relative humidity minimum, %	41.7	59.2	43.3	41.7

^1^THI = temperature-humidity index, calculated as THI = (0.8 × T) + [RH × (T – 14.4)] + 46.4, where T = temperature (°C), RH = relative humidity (decimal) [36].

^2^Week = sampling week (evaluation week) as defined in the Methods (weeks 3, 6, and 9 of the trial).

^3^Overall values were calculated from pooled hourly environmental records across sampling weeks 3, 6, and 9.

^4^Air temperature and relative humidity were measured inside the compost barn and reflect the in-barn microclimate under the cooling system (fans and sprinklers).

Among the three sampling periods, the greatest thermal challenge occurred during weeks 6 and 9, with THI > 72 for 37.7% and 40.1% of the time, respectively. Week 9 also had the highest mean THI (71.6 ± 1.5), as well as the highest average daily temperature (23.1 ± 1.3 °C) and the highest mean maximum temperature (29.5 ± 2.0 °C). In contrast, week 3 presented the mildest conditions, with the lowest mean THI (69.0 ± 2.2), lowest mean temperature (21.1 ± 1.5 °C), and the smallest proportion of hours above both THI thresholds (THI > 68: 56.1%; THI > 72: 19.8%).

**Fig 1:**
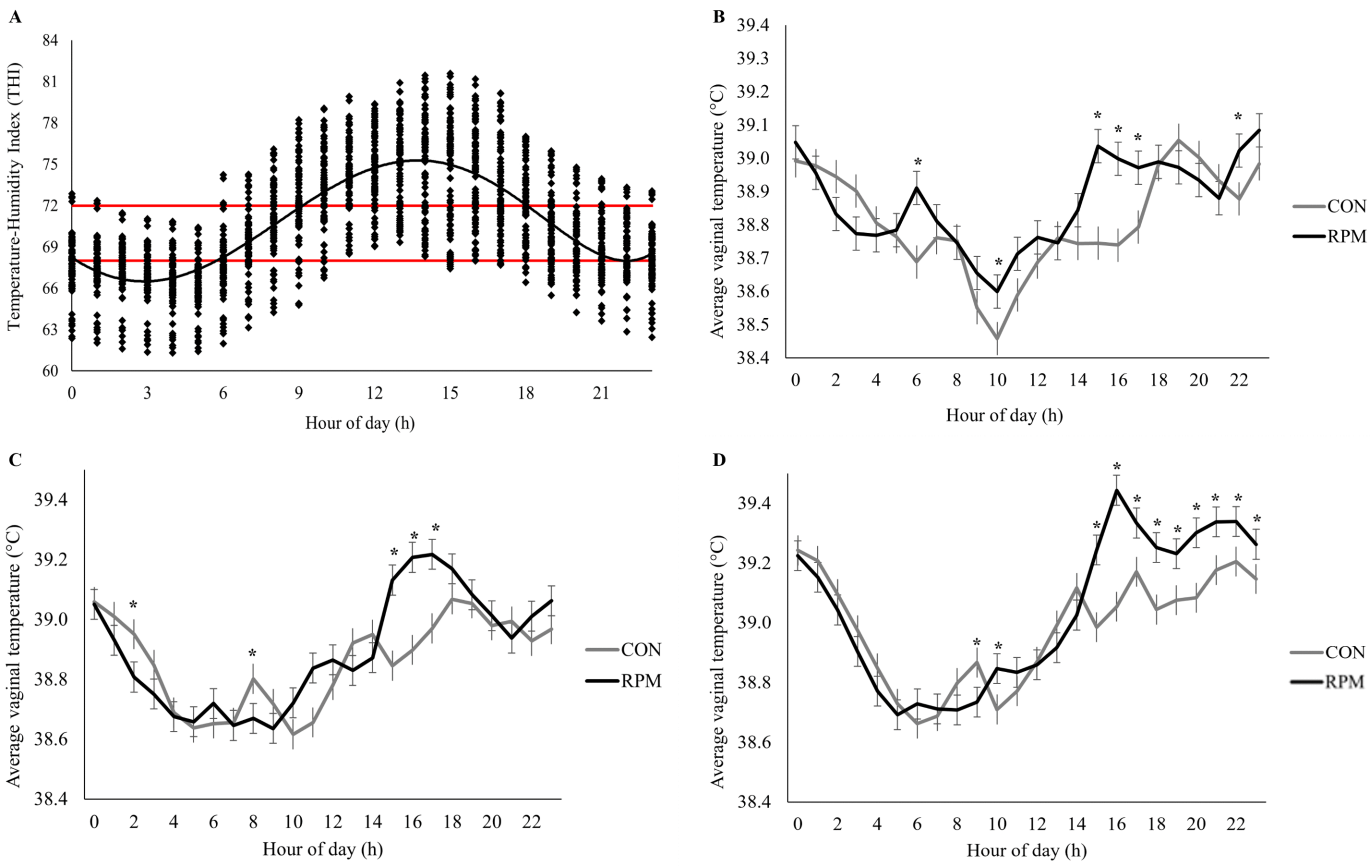
Temperature-humidity index (THI) and vaginal temperature profiles over a 24-h cycle during the sampling weeks (weeks 3, 6, and 9). (A) Variation of the temperature-humidity index (THI) recorded over a 24-h period. Each point represents an individual hourly measurement, and the solid line represents the smoothed trend across time. Data correspond to the combined observations from the sampling weeks (weeks 3, 6, and 9). Horizontal reference lines indicate THI thresholds of 68 and 72. THI was calculated as (0.8 × T) + [RH × (T − 14.4)] + 46.4, where T = temperature (°C) and RH = relative humidity expressed as a decimal (Mader et al., 2006). (B–D) Average vaginal temperature (°C) measured hourly over a 24-h period in lactating primiparous Holstein cows assigned to a control diet (CON; no added rumen-protected methionine) or supplemented with rumen-protected methionine (RPM; 0.75 g/kg diet DM) during sampling week 3 (B), week 6 (C), and week 9 (D) during the Brazilian summer. Bars represent means ± standard error of the mean (SEM). Asterisks (*) indicate time points with treatment differences (P ≤ 0.05).

The daily distribution of THI revealed a clear pattern, with the lowest values occurring between 00:00 and 06:00 h and the highest values between 12:00 and 16:00 h. During this peak period, most THI observations surpassed the threshold of 72, indicating consistent exposure to moderate-to-severe heat challenge in the afternoon hours (Fig 1A).

### Vaginal temperature and respiratory rate

Vaginal temperature and RR are presented in Table 3. No differences were detected between treatments for mean VT, maximum VT, or minimum VT. However, a significant treatment × time (week) × hour interaction was observed for VT (P < 0.001), as illustrated in Fig 1B–D. In week 3 (Fig 1B), cows supplemented with RPM exhibited higher VT than CON at 06:00, 10:00, 15:00, 16:00, 17:00, and 22:00 h (P < 0.05). In week 6 (Fig 1C), differences were detected at 02:00, 08:00, and from 15:00–17:00 h, with RPM cows presenting higher VT (P < 0.05). In week 9 (Fig 1D), RPM cows showed higher VT at 09:00 and 10:00, and from 15:00–23:00 h compared with CON (P < 0.05).

**Table 3:** Respiratory rate (n = 40) and vaginal temperature (n = 15) of primiparous Holstein cows supplemented (RPM) or not (CON) with rumen-protected methionine during summer.

Item	Treatment		SEM	*P*-value			
	CON	RPM		Trt	Week	Trt × Week	Trt × Week × Hour
Respiratory rate at 10:00 h, breaths/min	47.58	51.02	0.90	**0.009**	**< 0.001**	0.405	NA
Respiratory rate at 17:00 h, breaths/min	51.51	57.91	1.16	**< 0.001**	**< 0.001**	**0.024**	NA
Average vaginal temperature, °C	38.88	38.93	0.03	0.293	**< 0.001**	0.435	**< 0.001**
Minimum vaginal temperature, °C	38.75	38.79	0.03	0.302	**< 0.001**	0.638	**< 0.001**
Maximum vaginal temperature, °C	39.01	39.06	0.03	0.341	**< 0.001**	0.103	**< 0.001**

¹CON = control diet (no added rumen-protected methionine); RPM = rumen-protected methionine–supplemented diet (0.75 g/kg diet DM); SEM = standard error of the mean; Trt = treatment effect; Week = sampling week (weeks 3, 6, and 9); Hour = clock hour for vaginal temperature measurement (hourly values across 24 h); Trt × Week = treatment × sampling week interaction; Trt × Week × Hour = treatment × sampling week × measurement hour interaction. For respiratory rate, measurements were recorded at 10:00 and 17:00 h and the Trt × Week × Hour term was not applicable (NA). NA = not applicable (term not included in the model). Boldface P-values indicate statistical significance (P ≤ 0.05). Measurements were collected in lactating primiparous Holstein cows during the Brazilian summer.

Cows fed the RPM diet showed higher RR at 17:00 h (57.9 vs. 51.5 breaths/min; P < 0.001), and a tendency for greater RR at 10:00 h (51.0 vs. 47.6 breaths/min; P = 0.09), consistent with the observed interaction between treatment and time at 17:00 h (P = 0.024). During respiratory rate measurements (10:00 and 17:00 h), the proportion of cows with RR ≥ 60 breaths/min was higher in the afternoon and increased in weeks 6 and 9. At 17:00 h, RR ≥ 60 breaths/min occurred in 19.2%, 66.2%, and 60.3% of RPM cows and 5.0%, 21.3%, and 42.3% of CON cows in weeks 3, 6, and 9, respectively; at 10:00 h, corresponding proportions were 2.6%, 32.4%, and 39.7% (RPM) and 0.0%, 20.0%, and 30.8% (CON).

### Milk production and composition

**Fig 2:**
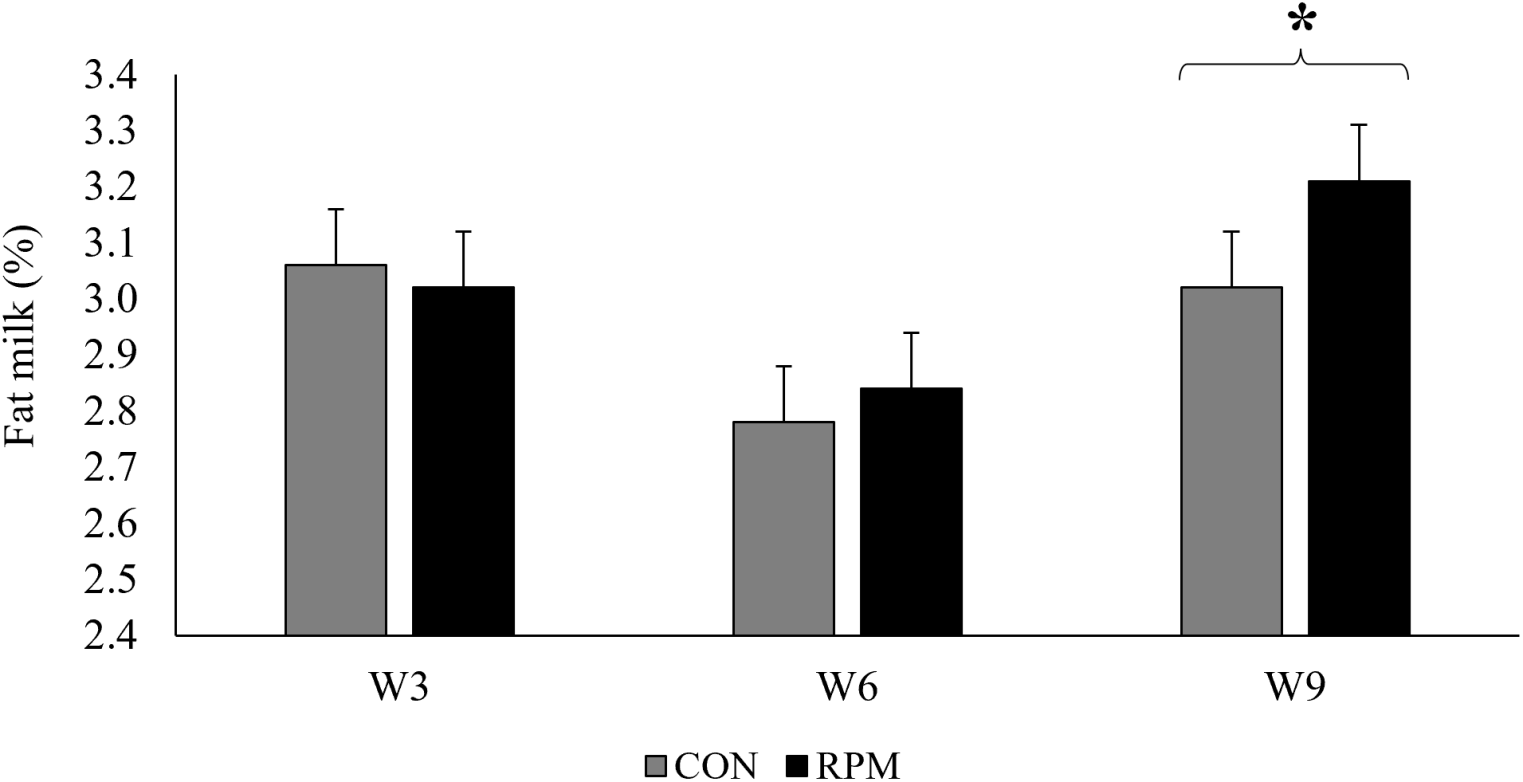
Milk fat concentration (%) during the sampling weeks (weeks 3, 6, and 9) in lactating primiparous Holstein cows assigned to a control diet (CON; no added rumen-protected methionine) or supplemented with rumen-protected methionine (RPM; 0.75 g/kg diet DM) during the Brazilian summer. Bars represent means ± standard error of the mean (SEM). Asterisk (*) above the bracket indicates a difference between treatments within that sampling week (P ≤ 0.05).

Milk yield and composition are presented in Table 4. Supplementation with RPM increased yields of milk (44.9 vs 42.9 kg/d, P < 0.01), protein (1464 vs 1414 g/d, P = 0.03), lactose (2109 vs 2001 g/d, P = 0.03) and total solids (5331 vs 5155 g/d, P = 0.03). Energy-corrected milk (42.1 vs 40.7 kg/d, P = 0.06) and casein yield (1156 vs 1121 g/d, P = 0.06) tended to be increased by the RPM treatment. Lactose concentration was reduced in RPM group compared with CON (4.71 vs. 4.76%; P = 0.01). Fat yield was not affected by RPM supplementation, but there was an interaction (P = 0.02) between treatment and week for fat content (Fig 2). For the RPM treatment, fat content was greater in week 9 compared to week 3, whereas in the control group it did not change significantly across weeks.

**Table 4:** Milk yield and composition of primiparous Holstein cows supplemented (RPM) or not (CON) with rumen-protected methionine during summer.

	Treatment		SEM	*P*-value		
Item	CON	RPM		Trt	Week	Trt × Week
Milk yield, kg/d	42.9	44.9	0.54	**0.001**	**0.001**	0.302
ECM^2^, kg/d	40.7	42.1	0.63	0.063	**< 0.001**	0.177
*Milk component yield, g/d*						
Fat	1269	1316	38.65	0.363	**< 0.001**	0.133
Protein	1414	1464	17.14	**0.032**	0.319	0.967
Lactose	2001	2109	23.24	**0.034**	**< 0.001**	0.528
Casein	1121	1156	14.61	0.063	0.253	0.950
Total solids	5155	5331	68.81	**0.038**	**< 0.001**	0.381
*Milk composition*						
Fat, %	2.95	3.02	0.10	0.767	**< 0.001**	**0.020**
Protein, %	3.30	3.27	0.02	0.248	**< 0.001**	0.097
Lactose, %	4.76	4.71	0.01	**0.010**	**0.006**	0.982
Casein, %	2.61	2.58	0.02	0.222	**< 0.001**	0.170
Total solids, %	11.99	11.97	0.10	0.855	**< 0.001**	0.109
MUN^3^, mg/dL	11.77	11.46	0.18	0.141	**< 0.001**	0.804
SCC^4^ (x 10³), cells/mL	132.17	178.82	30.23	0.594	0.958	0.378

^1^CON = control diet (no added rumen-protected methionine); RPM = rumen-protected methionine–supplemented diet (0.75 g/kg diet DM); SEM = standard error of the mean; Trt = treatment effect; Week = effect of sampling week (weeks 3, 6, and 9); Trt × Week = treatment × sampling week interaction. Boldface P-values indicate statistical significance (P ≤ 0.05). Measurements were collected in lactating primiparous Holstein cows during the Brazilian summer.

^2^Energy-corrected milk (ECM) was calculated as ECM (kg/d) = (0.327 × milk yield, kg) + (12.95 × fat yield, kg) + (7.2 × protein yield, kg), according to NASEM (2021).

^3^MUN = milk urea nitrogen.

^4^SCC = somatic cell count.

Milk fatty acid composition and yield are in Table 5. Supplementation with RPM did not affect FA profile for C14:0, C16:0, C18 and C18:1. De novo FA yield was not affected, as well as mixed FA and preformed FA.

**Table 5:** Milk fatty acid composition and yield of primiparous Holstein cows supplemented (RPM) or not (CON) with rumen-protected methionine during summer.

	Treatment		SEM	*P*-value		
Item	CON	RPM		Trt	Week	Trt × Week
*Fatty acids composition, g/100 g of milk*						
C14:0	0.268	0.282	0.010	0.242	**< 0.001**	0.116
C16:0	0.804	0.848	0.032	0.301	**< 0.001**	**0.049**
C18:0	0.220	0.222	0.009	0.853	**< 0.001**	**0.028**
C18:1	0.706	0.702	0.012	0.871	**< 0.001**	**0.007**
SFA^2^	1.654	1.732	0.069	0.491	**< 0.001**	**0.063**
UFA^3^	1.144	1.144	0.023	0.941	**< 0.001**	**0.024**
MUFA^4^	0.890	0.886	0.019	0.834	**< 0.001**	**0.027**
PUFA^5^	0.116	0.118	0.013	0.520	**< 0.001**	0.301
Short chain	0.142	0.151	0.014	0.397	**< 0.001**	0.130
Middle chain	1.290	1.357	0.047	0.299	**< 0.001**	**0.058**
Long chain	1.005	1.006	0.031	0.973	**< 0.001**	**0.005**
De novo	0.605	0.630	0.033	0.598	**< 0.001**	0.191
Mixed	0.806	0.827	0.036	0.591	**< 0.001**	**0.065**
Preformed	1.357	1.342	0.026	0.656	**< 0.001**	**0.019**
*Fatty acids yield, g/day*						
C14:0	115.21	122.50	4.39	0.200	**< 0.001**	0.192
C16:0	345.26	367.32	13.32	0.187	**< 0.001**	**0.092**
C18:0	94.78	95.67	3.89	0.864	**< 0.001**	**0.011**
C18:1	306.55	307.16	7.67	0.950	**< 0.001**	**0.001**
SFA^2^	710.92	749.88	27.50	0.297	**< 0.001**	**0.059**
UFA^3^	496.87	501.85	11.02	0.732	**< 0.001**	**0.013**
MUFA^4^	386.85	388.11	8.53	0.843	**< 0.001**	**0.013**
PUFA^5^	50.19	51.94	1.21	0.307	**< 0.001**	0.166
Short chain	59.90	64.81	5.97	0.331	**< 0.001**	0.109
Middle chain	554.38	588.97	19.99	0.156	**< 0.001**	0.106
Long chain	435.96	437.65	13.03	0.897	**< 0.001**	**0.002**
De novo	259.25	271.02	13.81	0.537	**< 0.001**	0.318
Mixed	348.00	357.05	15.32	0.586	0.160	**0.078**
Preformed	589.87	585.86	11.67	0.813	**< 0.001**	**0.019**

^1^CON = control diet (no added rumen-protected methionine); RPM = rumen-protected methionine–supplemented diet (0.75 g/kg diet DM); SEM = standard error of the mean; Trt = treatment effect; Week = effect of sampling week (weeks 3, 6, and 9); Trt × Week = treatment × sampling week interaction. Fatty acids are reported as carbon chain length: number of double bonds (e.g., C16:0). Boldface P-values indicate statistical significance (P ≤ 0.05). Measurements were collected in lactating primiparous Holstein cows during the Brazilian summer.

^2^SFA = saturated fatty acids.

^3^UFA = unsaturated fatty acids.

^4^MUFA = monounsaturated fatty acids.

^5^PUFA = polyunsaturated fatty acids.

However, a significant interaction between treatment and week was observed for preformed fatty acids (P = 0.02), and a trend was noted for mixed fatty acids (P = 0.08). In RPM-supplemented cows, preformed FA concentration increased in week 9 compared to week 3 (1.44 vs. 1.36%), and mixed FA also increased (0.87 vs. 0.79%). No significant temporal variation was observed in the control group.

### Biochemical, oxidative, immune, and hormonal parameters

Plasma concentrations of biochemical, oxidative stress, inflammatory, immune, and hormonal markers are presented in Table 6. Among the biochemical variables, plasma glucose was significantly lower in RPM compared with CON (39.3 vs. 43.2 mg/dL; P < 0.001), whereas other indicators such as albumin, alanine aminotransferase, high-density lipoprotein, and total cholesterol were not affected by treatment. A time effect was observed for plasma urea concentrations (P = 0.048), but no treatment effect or treatment × time interaction was detected.

**Table 6:** Plasma concentrations of biochemical, oxidative stress, inflammatory, immune, and hormonal parameters of primiparous Holstein cows supplemented (RPM) or not (CON) with rumen-protected methionine during summer.

	Treatment		SEM	*P*-value		
Item	CON	RPM		Trt	Week	Trt × Week
*Biochemical parameters*						
Albumin, mg/dL	3.40	3.42	0.02	0.363	0.921	0.218
Alanine aminotransferase (ALT), U/L	20.16	21.11	0.54	0.165	0.100	0.137
Aspartate aminotransferase (AST), U/L	111.31	108.00	4.64	0.345	0.115	0.448
Glucose, mg/dL	43.18	39.31	0.84	**< 0.001**	0.102	0.272
High-density lipoprotein (HDL), mg/dL	95.32	95.46	1.40	0.892	**< 0.001**	0.444
Total cholesterol, mg/dL	207.11	211.85	4.51	0.450	0.645	0.368
Urea, mg/dL	35.97	36.54	0.28	0.131	**0.048**	0.530
*Oxidative stress biomarkers*						
Ferric reducing antioxidant power (FRAP), µM	28.49	32.88	1.16	**0.006**	NA	NA
Glutathione peroxidase (GPX), U/mL	0.30	0.30	0.01	0.645	**< 0.001**	**0.046**
Malondialdehyde (MDA), nmol/mL	2.78	2.48	0.08	**0.007**	**< 0.001**	0.071
Superoxide dismutase (SOD), U/mL	44.28	43.21	2.07	0.712	**0.003**	0.797
*Inflammatory and immune markers*						
Haptoglobin, mg/dL	20.39	15.76	1.78	0.242	NA	NA
Immunoglobulin A (IgA), mg/dL	99.11	106.67	3.91	0.191	NA	NA
*Hormonal and metabolic regulation*						
Cortisol, ng/mL	12.27	13.64	0.87	0.315	NA	NA
Insulin, ng/mL	0.35	0.52	0.03	**< 0.001**	NA	NA
Non-esterified fatty acids (NEFA), mmol/L	0.25	0.27	0.01	0.478	NA	NA

^1^CON = control diet (no added rumen-protected methionine); RPM = rumen-protected methionine–supplemented diet (0.75 g/kg diet DM); SEM = standard error of the mean; Trt = treatment effect; Week = effect of sampling week (weeks 3, 6, and 9); Trt × Week = treatment × sampling week interaction. Week and Trt × Week are not applicable for variables measured only once during the experiment (week 9). NA = not applicable. Boldface P-values indicate statistical significance (P ≤ 0.05). Measurements were collected in lactating primiparous Holstein cows during the Brazilian summer.

**Fig 3:**
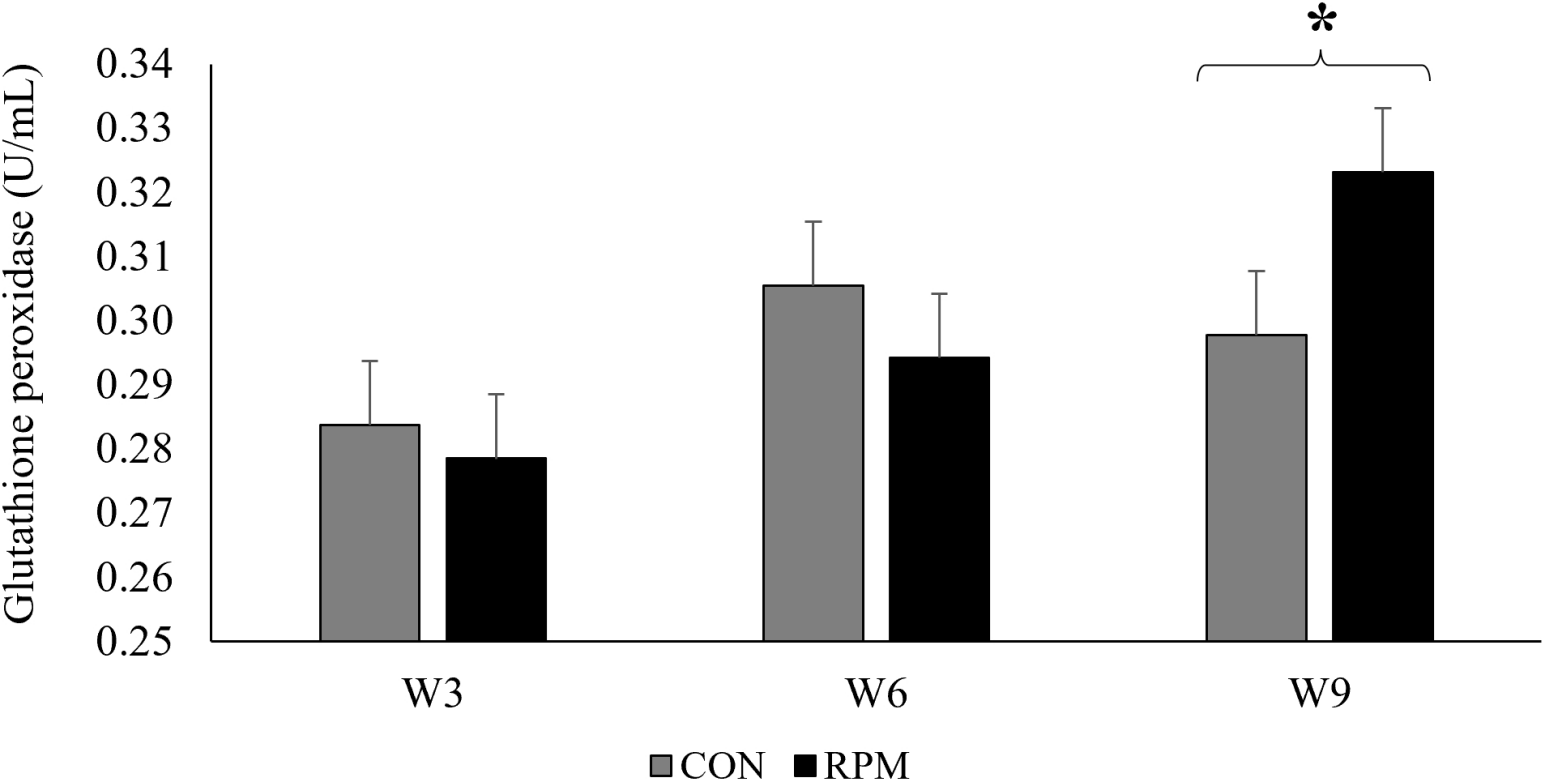
Plasma glutathione peroxidase activity (U/mL) during the sampling weeks (weeks 3, 6, and 9) in lactating primiparous Holstein cows assigned to a control diet (CON; no added rumen-protected methionine) or supplemented with rumen-protected methionine (RPM; 0.75 g/kg diet DM) during the Brazilian summer. Bars represent means ± standard error of the mean (SEM). Asterisk (*) above the bracket indicates a difference between treatments within that sampling week (P ≤ 0.05).

Regarding oxidative stress markers, RPM supplementation enhanced systemic antioxidant status, as evidenced by higher FRAP (32.9 vs. 28.5 µM; P = 0.006) and lower plasma MDA concentrations (2.48 vs. 2.78 nmol/mL; P = 0.007). No overall treatment effects were observed for GPX or SOD. However, GPX activity was affected by time (P < 0.001), and a treatment × time interaction was detected (P = 0.046), indicating distinct temporal responses between groups. As shown in Fig 3, GPX concentrations remained stable across weeks in CON cows, whereas RPM cows exhibited a progressive increase, reaching significantly higher values at week 9 compared with week 3 (P < 0.05), and also exceeding CON at that final time point.

Inflammatory and immune parameters were unaffected by treatment. Concentrations of haptoglobin and immunoglobulin A did not differ between groups.

Among hormonal variables, RPM-supplemented cows showed higher plasma insulin concentrations (0.52 vs. 0.35 ng/mL; P < 0.001), whereas cortisol and non-esterified fatty acids were unaffected.

## Discussion

In the present study, supplementation with rumen-protected methionine (RPM) during summer improved milk yield and the daily production of protein, lactose, and total solids in primiparous Holstein cows. RPM also enhanced systemic antioxidant capacity, as evidenced by higher FRAP and lower MDA, and increased plasma insulin concentrations. Although cows receiving RPM exhibited slightly higher respiratory rates and transient increases in vaginal temperature during hotter periods of the day, overall thermal status was not compromised. Collectively, these results indicate that RPM supplementation supported productive performance and metabolic resilience under sustained heat stress conditions.

Thermal comfort in dairy cows is commonly assessed using the THI, which combines ambient temperature and relative humidity. Recent evidence indicates that high genetic merit cows show signs of HS when THI exceeds 68 and experience marked production losses above 72 [41,42]. In the present study, mean THI was 70.6 ± 2.0, with values above 68 and 72 recorded during 68.3% and 32.5% of the monitored hours, respectively, indicating sustained heat load during the monitored sampling weeks and providing the basis for evaluating physiological and metabolic responses to RPM supplementation.

Despite the use of a cooling system, environmental monitoring showed sustained heat load, with THI frequently exceeding 68 and reaching high peak values. Consistent with this pattern, the distribution of respiratory rate observations indicated that a notable proportion of cows exceeded 60 breaths/min at the assessment times, particularly at 17:00 h in weeks 6 and 9. These observations suggest episodic physiological responses compatible with heat challenge during the hottest hours, even under heat-abatement management.

Physiological responses to heat differed between groups. Cows receiving RPM showed higher respiratory rates and transient increases in vaginal temperature during the afternoon hours, particularly in the later weeks. These effects may reflect increased metabolic activity associated with greater milk production rather than impaired thermoregulation, especially given the absence of differences in mean, minimum, or maximum 24-h vaginal temperatures between treatments [22]. The observed rises in vaginal temperature occurred predominantly during periods of higher ambient temperature and may indicate a greater internal heat load, possibly associated with intensified nutrient metabolism to support higher milk production.

In this study, cows supplemented with RPM produced more milk, accompanied by greater daily yields of protein, lactose, and total solids, along with a tendency for higher ECM, even under progressively increasing thermal challenge (mean THI: 69.0, 71.2, and 71.6 during weeks 3, 6, and 9, respectively). This pattern suggests that RPM helped support mammary secretory capacity under summer heat load.

It is important to note that individual DMI was not measured in this study, which limits definitive conclusions regarding the mechanisms underlying the productive responses observed. However, the improvement in the metabolic and oxidative status of RPM-supplemented cows, discussed later, suggests a more favorable physiological condition that may have influenced both nutrient use efficiency and potential voluntary intake. If such an increase in intake occurred, it is plausible that it contributed to part of the observed production response. The diet was formulated to meet the cows’ nutritional requirements, and the predicted energy supply was consistent with the observed production levels.

In the present experiment, RPM supplementation increased daily milk protein yield without affecting its concentration and showed a tendency to enhance casein synthesis, suggesting an increased synthetic capacity independent of the dilution effect caused by higher milk volume. Mechanistically, in addition to being a deficient AA for casein synthesis, methionine also acts as a metabolic signal. Through its derivative SAM, it has been linked to activation of the mTOR pathway and stimulates anabolic processes such as protein translation [43,44]. Recent evidence indicates that increasing post-ruminal methionine supply enhances mammary tissue mTOR phosphorylation (p-mTOR), linking it to greater milk protein synthesis in cows exposed to HS [18,43]. Thus, the increase in daily protein yield observed here may reflect both the provision of a deficient AA and the activation of signaling pathways that sustain protein synthesis during HS.

Additionally, HS has been consistently associated with a reduction in milk fat percentage and, in some cases, milk fat yield in dairy cows [25,45–47], although some studies have reported no significant changes [48,49]. Proposed mechanisms include decreased de novo fatty acid synthesis in the mammary gland, associated with reduced DMI and alterations in ruminal fermentation [50–52], as well as potential changes in hepatic triglyceride export [53]. In the present study, the temporal interaction observed for milk fat concentration, with higher values in RPM-supplemented cows in week 9, coincided with an increased proportion of preformed and mixed fatty acids in milk fat, suggesting maintenance of lipogenic capacity even under higher milk production. Although total fat yield remained unchanged, this pattern aligns with previous studies reporting variable effects of RPM on milk fat synthesis depending on basal diet and stage of lactation [54,55]. Methionine may influence milk fat synthesis through multiple pathways. As a lipotropic agent, it participates in the activation of the phosphatidylethanolamine methyltransferase (PEMT) pathway in the liver [56], which converts phosphatidylethanolamine into phosphatidylcholine (PC) using methyl groups donated by SAM. PC is the main phospholipid of very low-density lipoproteins (VLDL), and its increased availability promotes hepatic triglyceride export and enhances the supply of long-chain fatty acids to the mammary gland [21,57–59]. At the mammary level, methionine supplementation has been associated with increased expression of lipogenic genes such as PPARG and FASN, and with reduced abundance of miR-23a, a microRNA that directly represses lipogenic pathways, thereby favoring lipid synthesis [60]. Additionally, methionine acts as a metabolic signal via the mTOR pathway, which regulates key enzymes involved in fatty acid synthesis [61]. The specific impact of rumen-protected methionine supplementation on mammary lipogenesis under HS conditions warrants further investigation.

It is also important to highlight that this study was conducted with primiparous cows, which may respond differently to methionine supplementation compared with multiparous cows. In addition to sustaining lactation, primiparous animals must allocate nutrients to ongoing body growth, resulting in altered nutrient partitioning and greater sensitivity to dietary AA adequacy [62,63]. These animals often have lower DMI relative to their requirements [64], a reduced capacity to mobilize body reserves [65] and may respond more positively to increased metabolizable protein and methionine supply during early and mid-lactation [66,67]. Therefore, the productive responses observed in the present study may reflect a greater nutrient efficiency characteristic of primiparous cows when provided with balanced AA profiles.

Plasma biochemical and hormonal responses provided additional evidence of altered nutrient metabolism. Lower glucose concentrations can be explained by greater lactose production in RPM cows. Moreover, insulin has been demonstrated to be a potent regulator of milk protein synthesis by upregulating genes directly involved in protein translation, casein production, and AA uptake by the mammary gland *ex vivo* [68].

The liver plays a central role in the metabolic adaptation of high-producing cows under HS. Unlike the transition period, HS does not appear to promote pronounced mobilization of non-esterified fatty acids, as adipose tissue lipolysis is suppressed as a physiological strategy to reduce metabolic heat production, thereby exposing the liver to a lower lipid load [4,49]. In this study, serum ALT and AST concentrations remained within physiological ranges, suggesting preserved hepatic function. A temporal increase in HDL concentration was observed, with no treatment or treatment × time effects. HDL mediates reverse cholesterol transport and provides substrate for VLDL formation, which can then be exported to peripheral tissues, including the mammary gland. Although no direct association with treatment was detected, this temporal pattern may have supported lipid availability and contributed to the late increase in milk fat observed in RPM cows at the end of the experiment.

Heat stress exacerbates systemic oxidative stress by promoting an increased generation of reactive oxygen species (ROS) as a consequence of elevated energy demand and intensified mitochondrial activity [69]. This imbalance between ROS production and cellular antioxidant capacity compromises membrane integrity, leading to lipid peroxidation and structural damage. In the present study, a temporal pattern in glutathione peroxidase (GPX) activity was observed, with a significant treatment × time interaction. Although overall GPX means did not differ between groups, RPM-supplemented cows showed a progressive increase in enzyme activity throughout the weeks, reaching significantly higher values by week 9, whereas levels remained stable in the control group. This pattern paralleled the gradual increase in THI, suggesting that RPM supplementation promoted a more robust antioxidant response to escalating HS conditions. Methionine participates in one-carbon metabolism and, through the transsulfuration pathway, is converted into cysteine, the limiting AA for the synthesis of reduced glutathione, the primary substrate for GPX [70,71]. Thus, RPM supplementation may have enhanced GSH availability and strengthened the antioxidant system dependent on this molecule, increasing the capacity to neutralize lipid and hydrogen peroxides via GPX activity. Reflecting this improved antioxidant efficiency, total antioxidant capacity increased and plasma malondialdehyde, a classical marker of lipid peroxidation [72], decreased in RPM cows. The lower MDA concentration suggests reduced oxidative damage to membranes, while the higher FRAP values indicate greater systemic ability to neutralize ROS. Mechanistic studies in bovine mammary epithelial cells under hyperthermia support this interpretation. Methionine supplementation reduced lipid peroxidation (lower MDA), increased GPX, superoxide dismutase, and catalase activities, and decreased apoptosis [73]. *In vivo*, supplementation with a rumen-protected a methionine–zinc complex enhanced total antioxidant capacity and reduced MDA in cows under prolonged HS, reinforcing the potential of methionine to modulate redox balance, although the effect cannot be entirely separated from zinc in that study [14].

Lactating cows exposed to HS frequently develop a systemic inflammatory response characterized by increased proinflammatory cytokines such as interleukin-1β, interleukin-6, and tumor necrosis factor-α, which stimulate the synthesis of APP such as haptoglobin and lipopolysaccharide-binding protein [7,14,15,19,74]. The severity of this response can be assessed by the rise in circulating haptoglobin, a positive APP, and by the decrease in hepatic albumin synthesis, a negative APP [75]. In the present study, no significant differences between treatments were observed for haptoglobin or albumin. This result may indicate that although HS was sufficient to impact performance and oxidative metabolism, it did not reach the intensity required to induce systemic inflammation capable of markedly altering these APP. The respiration rates reached by the cows during the experiment strengthened this hypothesis, since they were always below 60 movements/min. Alternatively, RPM supplementation may have attenuated the inflammatory response, as suggested by the numeric lower mean haptoglobin concentration in supplemented cows, although not to a magnitude sufficient to achieve statistical significance. It is also important to note that the experimental design, with samples collected every three weeks, may not have captured transient peaks in haptoglobin or rapid declines in albumin concentration that can occur shortly after HS onset.

Collectively, these findings support the hypothesis that methionine supplementation enhances lactational performance through coordinated adjustments in nutrient metabolism, antioxidant capacity, and physiological resilience. The consistent productive benefits observed under HS conditions reinforce the potential of RPM to improve production efficiency in hot climates.

These findings should be interpreted in light of the study design and on-farm conditions. A key limitation is that dietary treatments were delivered at the pen level (one pen per treatment) and individual daily intake was not measured, which limits pen-level replication and warrants cautious interpretation of treatment effects based on cow-level outcomes. Nonetheless, conducting the trial under commercial summer conditions increases external validity by evaluating RPM responses in a real-world management setting and enabled the inclusion of a larger number of cows than would be feasible with individual feeding.

## Conclusions

Supplementation with RPM improved milk yield and increased milk protein, lactose, and total solids yields in primiparous Holstein cows during summer. These benefits likely reflect better AA balance and nutrient use. RPM also enhanced antioxidant capacity and increased plasma insulin, suggesting metabolic advantages. Although cows supplemented with RPM exhibited slightly higher vaginal temperatures and respiratory rates during the hottest hours, no detrimental changes or indices of concern in overall thermal status were observed. These findings support the use of methionine to improve performance and metabolic function in primiparous cows under summer heat load, during which THI remained > 68 for extended periods. Further research is warranted to elucidate the tissue-specific mechanisms involved and to evaluate long-term impacts on health and reproductive performance.

## References

[1] Laporta J, Ferreira FC, Ouellet V, Dado-Senn B, Almeida AK, De Vries A, et al. Late-gestation heat stress impairs daughter and granddaughter lifetime performance. J Dairy Sci. 2020;103(8):7555–68. doi: 10.3168/jds.2020-18154 32534930

[2] Roth Z. Reproductive physiology and endocrinology responses of cows exposed to environmental heat stress - Experiences from the past and lessons for the present. Theriogenology. 2020;155:150–6. doi: 10.1016/j.theriogenology.2020.05.040 32679440

[3] IPCC. Climate change 2021: the physical science basis. Contribution of Working Group I to the Sixth Assessment Report of the Intergovernmental Panel on Climate Change. Cambridge: Cambridge University Press; 2021 [cited 2024 Oct 15]. Available from: https://www.ipcc.ch/report/ar6/wg1/

[4] Baumgard LH, Rhoads RP Jr. Effects of heat stress on postabsorptive metabolism and energetics. Annu Rev Anim Biosci. 2013;1:311–37. doi: 10.1146/annurev-animal-031412-103644 25387022

[5] Bagath M, Krishnan G, Devaraj C, Rashamol VP, Pragna P, Lees AM, et al. The impact of heat stress on the immune system in dairy cattle: A review. Res Vet Sci. 2019;126:94–102. doi: 10.1016/j.rvsc.2019.08.011 31445399

[6] Becker CA, Collier RJ, Stone AE. Invited review: Physiological and behavioral effects of heat stress in dairy cows. J Dairy Sci. 2020;103(8):6751–70. doi: 10.3168/jds.2019-17929 32448584

[7] Ríus AG. Invited Review: Adaptations of protein and amino acid metabolism to heat stress in dairy cows and other livestock species. Applied Animal Science. 2019;35(1):39–48. doi: 10.15232/aas.2018-01805

[8] Horst EA, Mayorga EJ, Baumgard LH. International Symposium on Ruminant Physiology: Integrating our understanding of stress physiology. J Dairy Sci. 2025;108(7):7675–95. doi: 10.3168/jds.2024-25794 39947603

[9] Legrand A, Schütz KE, Tucker CB. Using water to cool cattle: behavioral and physiological changes associated with voluntary use of cow showers. J Dairy Sci. 2011;94(7):3376–86. doi: 10.3168/jds.2010-3901 21700023

[10] Armstrong DV. Heat stress interaction with shade and cooling. J Dairy Sci. 1994;77(7):2044–50. doi: 10.3168/jds.S0022-0302(94)77149-6 7929964

[11] Gao ST, Guo ZT, Baumgard LH, Ma L, Bu DP. Cooling ameliorates decreased milk protein metrics in heat-stressed lactating Holstein cows. J Dairy Sci. 2021;104(11):12139–52. doi: 10.3168/jds.2021-20451 34419281

[12] Knapp DM, Grummer RR. Response of lactating dairy cows to fat supplementation during heat stress. J Dairy Sci. 1991;74(8):2573–9. doi: 10.3168/jds.S0022-0302(91)78435-X 1655841

[13] Williams SRO, Milner TC, Garner JB, Moate PJ, Jacobs JL, Hannah MC, et al. Dietary Fat and Betaine Supplements Offered to Lactating Cows Affect Dry Matter Intake, Milk Production and Body Temperature Responses to an Acute Heat Challenge. Animals (Basel). 2021;11(11):3110. doi: 10.3390/ani11113110 34827840 PMC8614460

[14] Danesh Mesgaran M, Kargar H, Janssen R, Danesh Mesgaran S, Ghesmati A, Vatankhah A. Rumen-protected zinc-methionine dietary inclusion alters dairy cow performances, and oxidative and inflammatory status under long-term environmental heat stress. Front Vet Sci. 2022;9:935939. doi: 10.3389/fvets.2022.935939 36172606 PMC9510689

[15] Ruiz-González A, Suissi W, Baumgard LH, Martel-Kennes Y, Chouinard PY, Gervais R, et al. Increased dietary vitamin D3 and calcium partially alleviate heat stress symptoms and inflammation in lactating Holstein cows independent of dietary concentrations of vitamin E and selenium. J Dairy Sci. 2023;106(6):3984–4001. doi: 10.3168/jds.2022-22345 37164847

[16] Daddam JR, Daniel D, Kra G, Pelech I, Portnick Y, Moallem U, et al. Plant polyphenol extract supplementation affects performance, welfare, and the Nrf2-oxidative stress response in adipose tissue of heat-stressed dairy cows. J Dairy Sci. 2023;106(12):9807–21. doi: 10.3168/jds.2023-23549 37641328

[17] Abeyta MA, Al-Qaisi M, Horst EA, Mayorga EJ, Rodriguez-Jimenez S, Goetz BM, et al. Effects of dietary antioxidant supplementation on metabolism and inflammatory biomarkers in heat-stressed dairy cows. J Dairy Sci. 2023;106(2):1441–52. doi: 10.3168/jds.2022-22338 36543647

[18] Pate RT, Luchini D, Cant JP, Baumgard LH, Cardoso FC. Immune and metabolic effects of rumen-protected methionine during a heat stress challenge in lactating Holstein cows. J Anim Sci. 2021;99(12):skab323. doi: 10.1093/jas/skab323 34741611 PMC8648293

[19] Pate RT, Luchini D, Murphy MR, Cardoso FC. Effects of rumen-protected methionine on lactation performance and physiological variables during a heat stress challenge in lactating Holstein cows. J Dairy Sci. 2020;103(3):2800–13. doi: 10.3168/jds.2019-17305 31954567

[20] Davidson BD, Zambon AA, Guadagnin AR, Hoppmann A, Larsen GA, Sherlock DN, et al. Rumen-protected methionine supplementation during the transition period under artificially induced heat stress: Effects on cow-calf performance. J Dairy Sci. 2024;107(10):8654–69. doi: 10.3168/jds.2024-25028 39218072

[21] McFadden JW, Girard CL, Tao S, Zhou Z, Bernard JK, Duplessis M, et al. Symposium review: One-carbon metabolism and methyl donor nutrition in the dairy cow. J Dairy Sci. 2020;103(6):5668–83. doi: 10.3168/jds.2019-17319 32278559

[22] Zhou Z, Vailati-Riboni M, Trevisi E, Drackley JK, Luchini DN, Loor JJ. Better postpartal performance in dairy cows supplemented with rumen-protected methionine compared with choline during the peripartal period. J Dairy Sci. 2016;99(11):8716–32. doi: 10.3168/jds.2015-10525 27638261

[23] Osorio JS, Ji P, Drackley JK, Luchini D, Loor JJ. Supplemental Smartamine M or MetaSmart during the transition period benefits postpartal cow performance and blood neutrophil function. J Dairy Sci. 2013;96(10):6248–63. doi: 10.3168/jds.2012-5790 23910549

[24] Batistel F, Arroyo JM, Bellingeri A, Wang L, Saremi B, Parys C, et al. Ethyl-cellulose rumen-protected methionine enhances performance during the periparturient period and early lactation in Holstein dairy cows. J Dairy Sci. 2017;100(9):7455–67. doi: 10.3168/jds.2017-12689 28711252

[25] Ouellet V, Cabrera VE, Fadul-Pacheco L, Charbonneau É. The relationship between the number of consecutive days with heat stress and milk production of Holstein dairy cows raised in a humid continental climate. J Dairy Sci. 2019;102(9):8537–45. doi: 10.3168/jds.2018-16060 31255266

[26] Lovarelli D, Minozzi G, Arazi A, Guarino M, Tiezzi F. Effect of extended heat stress in dairy cows on productive and behavioral traits. Animal. 2024;18(3):101089. doi: 10.1016/j.animal.2024.101089 38377809

[27] Lees AM, Sejian V, Wallage AL, Steel CC, Mader TL, Lees JC, et al. The Impact of Heat Load on Cattle. Animals (Basel). 2019;9(6):322. doi: 10.3390/ani9060322 31174286 PMC6616461

[28] Castro-Montoya J, Corea EE. Heat stress effects in primiparous and multiparous lactating crossbred cows under a warm environment and their responses to a cooling treatment. Animal Production Science. 2021;61(6):577–85. doi: 10.1071/an19398

[29] Kononoff PJ, Hanford KJ. Technical note: estimating statistical power of mixed models used in dairy nutrition experiments. J Dairy Sci. 2006;89(10):3968–71. doi: 10.3168/jds.S0022-0302(06)72439-0 16960072

[30] NASEM (National Academies of Sciences, Engineering, and Medicine). Nutrient requirements of dairy cattle. 8th rev. ed. Washington, D.C.: National Academies Press; 2021. 38386771

[31] AOAC International. Official methods of analysis. 19th ed. Gaithersburg: AOAC International; 2012. Method 942.05, Method 920.39.

[32] AOAC International. Official methods of analysis. 16th ed. Arlington: AOAC International; 1996. Method 942.05, Method 954.01, Method 973.18.

[33] Mertens DR, Allen M, Carmany J, Clegg J, Davidowicz A, Drouches M, et al. Gravimetric Determination of Amylase-Treated Neutral Detergent Fiber in Feeds with Refluxing in Beakers or Crucibles: Collaborative Study. Journal of AOAC International. 2002;85(6):1217–40. doi: 10.1093/jaoac/85.6.1217 12477183

[34] Goering HK, Van Soest PJ. Forage fiber analyses (apparatus, reagents, procedures, and some applications). Agric. Handb. No. 379. Washington, D.C.: U.S. Department of Agriculture; 1970.

[35] Hall MB. Determination of Starch, Including Maltooligosaccharides, in Animal Feeds: Comparison of Methods and a Method Recommended for AOAC Collaborative Study. Journal of AOAC International. 2009;92(1):42–9. doi: 10.1093/jaoac/92.1.42 19382561

[36] Mader TL, Davis MS, Brown-Brandl T. Environmental factors influencing heat stress in feedlot cattle. J Anim Sci. 2006;84(3):712–9. doi: 10.2527/2006.843712x 16478964

[37] Esterbauer H, Cheeseman KH. Determination of aldehydic lipid peroxidation products: malonaldehyde and 4-hydroxynonenal. Methods Enzymol. 1990;186:407–21. doi: 10.1016/0076-6879(90)86134-h 2233308

[38] Marklund S, Marklund G. Involvement of the superoxide anion radical in the autoxidation of pyrogallol and a convenient assay for superoxide dismutase. Eur J Biochem. 1974;47(3):469–74. doi: 10.1111/j.1432-1033.1974.tb03714.x 4215654

[39] Flohé L, Günzler WA. Assays of glutathione peroxidase. Methods Enzymol. 1984;105:114–21. doi: 10.1016/s0076-6879(84)05015-1 6727659

[40] Weber K, Osborn M. The Reliability of Molecular Weight Determinations by Dodecyl Sulfate-Polyacrylamide Gel Electrophoresis. Journal of Biological Chemistry. 1969;244(16):4406–12. doi: 10.1016/s0021-9258(18)94333-4 5806584

[41] Collier RJ, Hall LW, Rungrueng S, Zimbleman RB. Quantifying heat stress and its impact on metabolism and performance. In: Proceedings of the 24th Annual Southwest Nutrition & Management Conference. Tempe, AZ. 2012. p.74–84.

[42] Tao S, Orellana RM, Weng X, Marins TN, Dahl GE, Bernard JK. Symposium review: The influences of heat stress on bovine mammary gland function. J Dairy Sci. 2018;101(6):5642–54. doi: 10.3168/jds.2017-13727 29331468

[43] Coleman DN, Totakul P, Onjai-Uea N, Aboragah A, Jiang Q, Vailati-Riboni M, et al. Rumen-protected methionine during heat stress alters mTOR, insulin signaling, and 1-carbon metabolism protein abundance in liver, and whole-blood transsulfuration pathway genes in Holstein cows. J Dairy Sci. 2022;105(9):7787–804. doi: 10.3168/jds.2021-21379 35879168

[44] Arriola Apelo SI, Knapp JR, Hanigan MD. Invited review: Current representation and future trends of predicting amino acid utilization in the lactating dairy cow. J Dairy Sci. 2014;97(7):4000–17. doi: 10.3168/jds.2013-7392 24767883

[45] Gao ST, Guo J, Quan SY, Nan XM, Fernandez MVS, Baumgard LH, et al. The effects of heat stress on protein metabolism in lactating Holstein cows. J Dairy Sci. 2017;100(6):5040–9. doi: 10.3168/jds.2016-11913 28390717

[46] Kadzere CT, Murphy MR, Silanikove N, Maltz E. Heat stress in lactating dairy cows: a review. Livestock Production Science. 2002;77(1):59–91. doi: 10.1016/s0301-6226(01)00330-x

[47] Hou Y, Zhang L, Dong RY, Liang MY, Lu Y, Sun XQ, et al. Comparing responses of dairy cows to short-term and long-term heat stress in climate-controlled chambers. J Dairy Sci. 2021;104(2):2346–56. doi: 10.3168/jds.2020-18946 33272576

[48] Rhoads ML, Rhoads RP, VanBaale MJ, Collier RJ, Sanders SR, Weber WJ, et al. Effects of heat stress and plane of nutrition on lactating Holstein cows: I. Production, metabolism, and aspects of circulating somatotropin. J Dairy Sci. 2009;92(5):1986–97. doi: 10.3168/jds.2008-1641 19389956

[49] Wheelock JB, Rhoads RP, Vanbaale MJ, Sanders SR, Baumgard LH. Effects of heat stress on energetic metabolism in lactating Holstein cows. J Dairy Sci. 2010;93(2):644–55. doi: 10.3168/jds.2009-2295 20105536

[50] Bauman DE, Brown RE, Davis CL. Pathways of fatty acid synthesis and reducing equivalent generation in mammary gland of rat, sow, and cow. Arch Biochem Biophys. 1970;140(1):237–44. doi: 10.1016/0003-9861(70)90028-7 4394114

[51] Liu Z, Ezernieks V, Wang J, Arachchillage NW, Garner JB, Wales WJ, et al. Heat Stress in Dairy Cattle Alters Lipid Composition of Milk. Sci Rep. 2017;7(1):961. doi: 10.1038/s41598-017-01120-9 28424507 PMC5430412

[52] Urrutia NL, Harvatine KJ. Acetate Dose-Dependently Stimulates Milk Fat Synthesis in Lactating Dairy Cows. J Nutr. 2017;147(5):763–9. doi: 10.3945/jn.116.245001 28331053

[53] Basiricò L, Morera P, Lacetera N, Ronchi B. Down-regulation of hepatic ApoB100 expression during hot season in transition dairy cows. Livest Sci. 2011;137:49–57.

[54] Zanton GI, Bowman GR, Vázquez-Añón M, Rode LM. Meta-analysis of lactation performance in dairy cows receiving supplemental dietary methionine sources or postruminal infusion of methionine. J Dairy Sci. 2014;97(11):7085–101. doi: 10.3168/jds.2014-8220 25242429

[55] Patton RA. Effect of rumen-protected methionine on feed intake, milk production, true milk protein concentration, and true milk protein yield, and the factors that influence these effects: a meta-analysis. J Dairy Sci. 2010;93(5):2105–18. doi: 10.3168/jds.2009-2693 20412926

[56] Zhou Z, Garrow TA, Dong X, Luchini DN, Loor JJ. Hepatic Activity and Transcription of Betaine-Homocysteine Methyltransferase, Methionine Synthase, and Cystathionine Synthase in Periparturient Dairy Cows Are Altered to Different Extents by Supply of Methionine and Choline. J Nutr. 2017;147(1):11–9. doi: 10.3945/jn.116.240234 27881594

[57] Bionaz M, Loor JJ. Gene networks driving bovine milk fat synthesis during the lactation cycle. BMC Genomics. 2008;9:366. doi: 10.1186/1471-2164-9-366 18671863 PMC2547860

[58] Zom RLG, van Baal J, Goselink RMA, Bakker JA, de Veth MJ, van Vuuren AM. Effect of rumen-protected choline on performance, blood metabolites, and hepatic triacylglycerols of periparturient dairy cattle. J Dairy Sci. 2011;94(8):4016–27. doi: 10.3168/jds.2011-4233 21787937

[59] Myers WA, Rico JE, Davis AN, Fontoura ABP, Dineen MJ, Tate BN, et al. Effects of abomasal infusions of fatty acids and one-carbon donors on hepatic ceramide and phosphatidylcholine in lactating Holstein dairy cows. J Dairy Sci. 2019;102(8):7087–101. doi: 10.3168/jds.2018-16200 31178188

[60] Salama AAK, Duque M, Wang L, Shahzad K, Olivera M, Loor JJ. Enhanced supply of methionine or arginine alters mechanistic target of rapamycin signaling proteins, messenger RNA, and microRNA abundance in heat-stressed bovine mammary epithelial cells in vitro. J Dairy Sci. 2019;102(3):2469–80. doi: 10.3168/jds.2018-15219 30639019

[61] Li B, Khan MZ, Khan IM, Ullah Q, Cisang Z-M, Zhang N, et al. Genetics, environmental stress, and amino acid supplementation affect lactational performance via mTOR signaling pathway in bovine mammary epithelial cells. Front Genet. 2023;14:1195774. doi: 10.3389/fgene.2023.1195774 37636261 PMC10448190

[62] Adachi N, Kusuhara T, Nonaka I, Terada F. Effect of Close-up Dry Period Protein Level on Preparturiental Nitrogen Balance and Lactating Performance of Primigravid and Multiparous Holstein Cows. Asian Australas J Anim Sci. 2006;19(6):831–6. doi: 10.5713/ajas.2006.831

[63] Husnain A, Santos JEP. Meta-analysis of the effects of prepartum dietary protein on performance of dairy cows. J Dairy Sci. 2019;102(11):9791–813. doi: 10.3168/jds.2018-16043 31495616

[64] Bell AW. Regulation of organic nutrient metabolism during transition from late pregnancy to early lactation. J Anim Sci. 1995;73(9):2804–19. doi: 10.2527/1995.7392804x 8582872

[65] Drackley JK, Overton TR, Douglas GN. Adaptations of Glucose and Long-Chain Fatty Acid Metabolism in Liver of Dairy Cows during the Periparturient Period. Journal of Dairy Science. 2001;84:E100–12. doi: 10.3168/jds.s0022-0302(01)70204-4

[66] Colmenero JJO, Broderick GA. Effect of dietary crude protein concentration on milk production and nitrogen utilization in lactating dairy cows. J Dairy Sci. 2006;89(5):1704–12. doi: 10.3168/jds.S0022-0302(06)72238-X 16606741

[67] Lee C, Hristov AN, Cassidy TW, Heyler KS, Lapierre H, Varga GA, et al. Rumen-protected lysine, methionine, and histidine increase milk protein yield in dairy cows fed a metabolizable protein-deficient diet. J Dairy Sci. 2012;95(10):6042–56. doi: 10.3168/jds.2012-5581 22863104

[68] Menzies KK, Lefèvre C, Macmillan KL, Nicholas KR. Insulin regulates milk protein synthesis at multiple levels in the bovine mammary gland. Funct Integr Genomics. 2009;9(2):197–217. doi: 10.1007/s10142-008-0103-x 19107532

[69] Burhans WS, Rossiter Burhans CA, Baumgard LH. Invited review: Lethal heat stress: The putative pathophysiology of a deadly disorder in dairy cattle. J Dairy Sci. 2022;105(5):3716–35. doi: 10.3168/jds.2021-21080 35248387

[70] Brosnan JT, Brosnan ME. The sulfur-containing amino acids: an overview. J Nutr. 2006;136(6 Suppl):1636S-1640S. doi: 10.1093/jn/136.6.1636S 16702333

[71] Loor JJ, Lopreiato V, Palombo V, D’Andrea M. Physiological impact of amino acids during heat stress in ruminants. Anim Front. 2023;13(5):69–80. doi: 10.1093/af/vfad052 37841758 PMC10575319

[72] Sehirli O, Tozan A, Omurtag GZ, Cetinel S, Contuk G, Gedik N, et al. Protective effect of resveratrol against naphthalene-induced oxidative stress in mice. Ecotoxicol Environ Saf. 2008;71(1):301–8. doi: 10.1016/j.ecoenv.2007.08.023 18261796

[73] Han Z-Y, Mu T, Yang Z. Methionine protects against hyperthermia-induced cell injury in cultured bovine mammary epithelial cells. Cell Stress Chaperones. 2015;20(1):109–20. doi: 10.1007/s12192-014-0530-7 25108357 PMC4255250

[74] Fontoura ABP, Javaid A, Sáinz de la Maza-Escolà V, Salandy NS, Fubini SL, Grilli E, et al. Heat stress develops with increased total-tract gut permeability, and dietary organic acid and pure botanical supplementation partly restores lactation performance in Holstein dairy cows. J Dairy Sci. 2022;105(9):7842–60. doi: 10.3168/jds.2022-21820 35931486

[75] Bertoni G, Trevisi E, Han X, Bionaz M. Effects of inflammatory conditions on liver activity in puerperium period and consequences for performance in dairy cows. J Dairy Sci. 2008;91(9):3300–10. doi: 10.3168/jds.2008-0995 18765589

